# Review of Methods for Studying Viruses in the Environment and Organisms

**DOI:** 10.3390/v17010086

**Published:** 2025-01-11

**Authors:** Xinyue Wang, Tong Ma, Zhiyuan Chen, Yang Liu, Kexin Wang, Guangxiu Liu, Kesheng Li, Tuo Chen, Gaosen Zhang, Wei Zhang, Binglin Zhang

**Affiliations:** 1 Key Laboratory of Ecological Safety and Sustainable Development in Arid Lands, Northwest Institute of Eco-Environment and Resources, Chinese Academy of Sciences, Lanzhou 730000, China; wangxinyue22@mails.ucas.ac.cn (X.W.); chenzhiyuan22@mails.ucas.ac.cn (Z.C.); liugx@lzb.ac.cn (G.L.); gaosenzhang@hotmail.com (G.Z.); ziaoshen@163.com (W.Z.); 2 Key Laboratory of Extreme Environmental Microbial Resources and Engineering, Lanzhou 730000, China; matong8882024@163.com (T.M.); liuyang21@nieer.ac.cn (Y.L.); 222083002012@lut.edu.cn (K.W.); chentuo@lzb.ac.cn (T.C.); 3 University of Chinese Academy of Sciences, No. 19A Yuquan Road, Beijing 100049, China; 4 School of Petrochemical Technology, Lanzhou University of Technology, Lanzhou 730050, China; 5 State Key Laboratory of Cryospheric Science and Frozen Soil Engineering, Northwest Institute of Eco-Environment and Resources, Chinese Academy of Sciences, Lanzhou 730000, China; 6 Lanzhou Yahua Biotechnology Company, Lanzhou 730050, China; likesheng63@hotmail.com

**Keywords:** virus communities, environmental samples, organism samples, research methods

## Abstract

Recent decades have seen growing attention on viruses in the environment and their potential impacts as a result of global epidemics. Due to the diversity of viral species along with the complexity of environmental and host factors, virus extraction and detection methods have become key for the study of virus ecology. This review systematically summarises the methods for extracting and detecting pathogens from different environmental samples (e.g., soil, water, faeces, air) and biological samples (e.g., plants, animals) in existing studies, comparing their similarities and differences, applicability, as well as the advantages and disadvantages of each method. Additionally, this review discusses future directions for research in this field. The aim is to provide a theoretical foundation and technical reference for virus ecology research, facilitating further exploration and applications in this field.

## 1. Introduction

### 1.1. Role of Viruses in the Environment and Organisms

Viruses are widely found in a variety of environments, where they play an important role. They form the foundation of the Earth’s ecological pyramid and significantly impact bacterial diversity and population structure. By infecting and killing bacteria, viruses participate in and influence the material and energy cycles within ecosystems. For example, viruses in oceans, such as those from the Caudovirales and Microviridae phage families, harbour genes that enable them to manipulate infected bacteria to process sulphur-containing compounds and participate in various cellular processes, such as photosynthesis [1]. The unique structural characteristics and small size of viruses allow for them to survive in a wide range of soils [2], facilitating virus transmission between plants and soil. For example, the cucumber green mottled mosaic virus (CGMMV) of the genus *Tobamovirus* infects grafted watermelons and spreads through pruning, irrigation, and other agricultural practices [3].

In addition to habitat-based viruses, there are also viruses that are transmitted through living organisms, such as the zoonotic snowshoe hare virus (SSHV), an arbovirus that is transmitted between small mammals and mosquitoes [4]. Rabies is another of the world’s deadliest zoonotic diseases, with bats serving as the primary host and source of transmission to humans and livestock [5].

Detecting virus–host (VH) interactions is important for in-depth studies of virus transmission between habitats. Since most cellular functions are supported by proteins, by studying VH interactions, we can more quickly find antiviral targets that play important functions in the viral life cycle [6]. Some convenient and fast mathematical modelling methods for the analysis of VH interactions have also emerged, such as dynamic optimisation, evolutionary game theory, and the modelling of spatial phenomena, among other computational methods [7]. However, the presence of viruses is not solely destructive to habitats; they also have positive effects. For example, viruses contribute to the release and cycling of carbon in the oceans by infecting and killing bacteria, which plays a role in maintaining the productivity of marine ecosystems and regulating global temperatures [8]. Additionally, viral infections promote inter-gene exchange, where viruses transfer genes between different hosts, facilitating horizontal gene transfer—an important mechanism in the evolution of organisms. For example, adenoviruses have been used as vectors for gene transfer and as therapeutic tools for cancer, owing to their unique envelope-free DNA structure. Adenoviral vectors can be modified to make them replication defective for use in gene therapy [9]. Similarly, viruses have a profound impact on human society, not only by causing human diseases but also through their applications in the fields of medicine and biotechnology. Currently, pathogens are often used as tools for vaccine development and gene therapy, aiding humans in combating certain diseases [10].

### 1.2. Current Status of Virus Research in the Environment and Organisms

**Figure 1:**
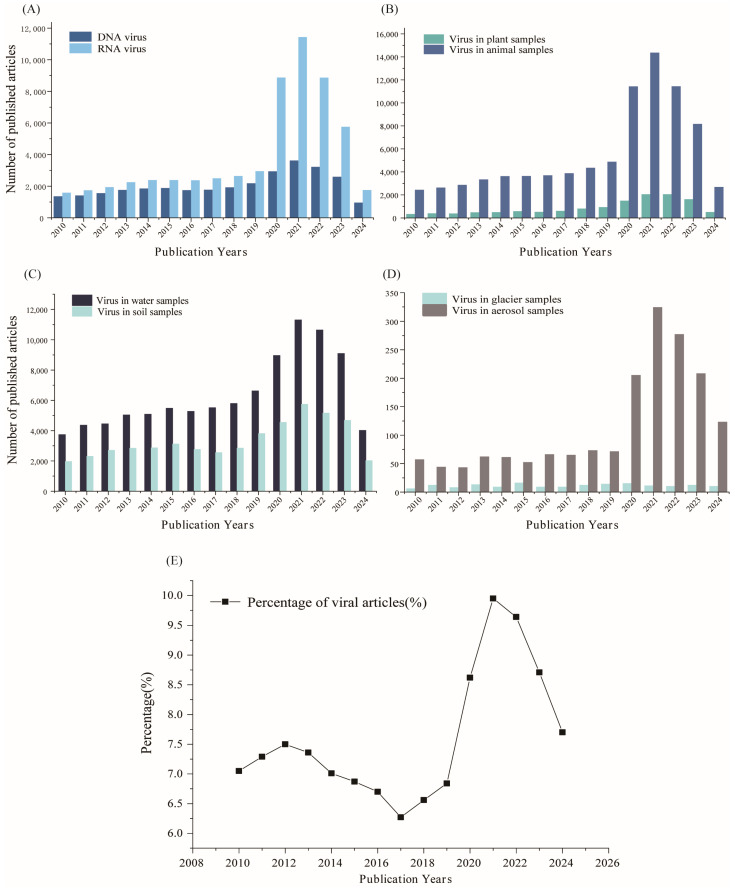
Literature statistics of studies published between 2010 and 2024 showing the number of different sample types, virus types, and virus-related articles as a percentage of total publications in the field of biology. (**A**) Trends in the number of articles published on DNA viruses and RNA viruses. (**B**) Trends in the amount of literature on virus research in plant samples and animal samples. (**C**) Trends in the amount of literature on virus studies in water and soil samples. (**D**) Trends in the amount of literature on viral studies in glacial and aerosol samples. (**E**) Trends in the number of virus-related articles as a percentage of all articles published in biology, 2010–2024.

According to the Baltimore virus classification system, the viruses discovered can be categorised into DNA viruses and RNA viruses. DNA viruses are further classified into single-stranded DNA viruses, double-stranded DNA viruses, and double-stranded DNA reverse transcription viruses, while RNA viruses are divided into double-stranded RNA viruses, positive-stranded RNA viruses, negative-stranded RNA viruses, and single-stranded RNA reverse transcription viruses [11]. Previously, there has been relatively limited research on RNA viruses due to the inherent instability of RNA, which makes these viruses highly susceptible to degradation by contaminants during the research process. This has contributed to fewer studies. Figure 1A shows the number of articles published on different virus types between 2010 and 2025. As can be seen, the emergence of a novel coronavirus (a single-stranded, positive-stranded RNA virus) in 2019 has led to an increasing interest in RNA viruses [12]. In addition, the literature on RNA virus research grows exponentially from 2020 onwards.

Current research on viruses, whether in the environment or in organisms, primarily focuses on virus–host interactions; the distribution of viruses in natural environments; and their evolution, survival patterns, and modes of transmission. The outbreak of the novel coronavirus (SARS-CoV-2, the agent of COVID-19) has sparked widespread interest in the virus–host–environment triad, driving research advancements in viral ecology [13,14,15]. As illustrated in Figure 1A–D, the three years following the onset of the global outbreak of COVID-19 in late 2020 and the subsequent lifting of full control measures at the beginning of 2023 saw a sharp rise in the number of articles on various virus classes worldwide. The number of such articles increased by as much as three- to four-fold compared to previous years. Although the number of related articles has decreased compared with the previous years, the volume of publications in the past two years remains high. This indicates a sustained increase in interest in viruses, especially in the study of some viruses affecting plants and animals. Polar, glacial, and permafrost environments, which are significantly affected by climate change, are research hotspots but still have fewer related viral studies. As shown in Figure 1D, fewer than 200 articles were published on viruses in glaciers between 2010 and 2024. This limited research is partly due to the challenges of sampling in glacial environments and partly due to the relatively low biomass compared to other environments, which renders conventional detection techniques less effective. This also suggests significant potential for future research on viruses in glacial environments. Figure 1E illustrates the changing trend in the number of virus-related articles as a percentage of all articles published in the biological community from 2010 to 2024, with the same trend evident from 2019 onwards, suggesting heightened concern regarding the presence of viruses in habitats following the COVD-19 outbreak.

**Figure 2:**
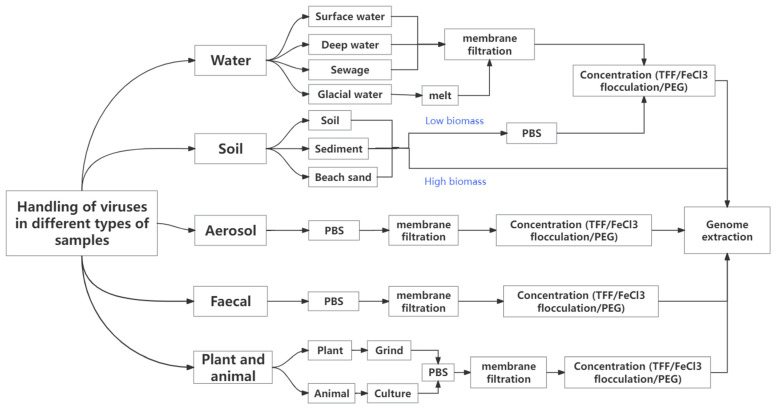
Flowchart illustrating pre-treatment of viruses for different types of samples.

This review provides a summary of the pre-treatment methods used in previous virus studies, as shown in Figure 2. There is little difference in the principles of the methods used to study viruses in the various matrices, which involve extracting the virus particles from the samples and then analysing their genomes. However, the methods for collecting and processing different types of samples are different. For instance, the extraction of viral particles from water samples requires filtration and concentration prior to nucleic acid extraction, while solid samples must be resuspended in PBS (phosphate buffered saline) buffer, followed by filtration and concentration for further analysis. During the sample collection step, the operational methods may vary, even for the same type of samples. For example, some surface water samples can be collected directly using sterile bags or bottles, whereas deep water samples necessitate specialised water samplers. This review aims to summarise and analyse the differences in viral research methods across various sample types.

### 1.3. Methodological Issues in Virus Research in the Current Environment

In the past, there have been a number of traditional viral particle assays, such as the plaque forming units (PFU) method [16], the 50% tissue culture infective dose (TCID_50_) method [17], and the real-time fluorescence quantitative PCR (qPCR) method [18,19], and of these three methods, while the qPCR method is rapid and sensitive, it does not ensure quantification of infectious virus particles only. However, there is a proliferation of emerging methods for viral titration. For example, the fluorescence focus assay (FFA) can detect and titrate viral infectivity based on the binding of specific antibodies to viral antigens, especially for viruses that fail to form empty spots using classical crystal violet or neutral red staining [20,21]. There is also the enzyme-linked immunosorbent assay (ELISA), which is fast, simple, specific, efficient, and similar in accuracy and sensitivity to traditional methods [22]. Currently, droplet digital PCR (ddPCR) is also highly used, which is highly accurate and allows for absolute quantification of target virus particles in samples without the need to prepare a standard curve [23,24]. Currently, there are still major constraints in the enrichment and pre-processing of viruses for research. By summarising and comparing different processing methods, this review aims to facilitate researchers to choose more efficient methods for virus research and make improvements.

## 2. Methods for Studying Viruses in Different Types of Samples

### 2.1. Methods for Studying Viruses in Water Samples

During the COVID-19 pandemic, viral particle levels in water have been reported to reach 4.60 ± 2.50 × 10^6^ GC/μL [25]. The concentration of viruses in different water bodies can vary widely, necessitating pre-experimentation to determine the appropriate sampling volume prior to analysis, especially in water bodies where viruses exist in diverse species and in large quantities. For example, viruses that infect humans are commonly found in domestic water and municipal wastewater [26,27,28,29,30,31], and fish-associated viruses are often detected in aquatic environments where fish are present [32,33,34,35].

#### 2.1.1. Water Sampling Methods

The process of collecting surface water is relatively straightforward. To enrich the water samples for virus particles, it is necessary to filter the samples to obtain a sediment containing microorganisms. Subsequently, the genome is extracted from the obtained sediment. Water samples are generally collected in sterile bags [36] or sterile bottles [37,38,39,40,41] at appropriate depths below the water surface. The samples are then preserved at appropriate temperatures and transported to the laboratory in a timely manner. The methods for collecting water samples vary depending on the depth of the source. As shown in Table 1, deep water from environments such as oceans and lakes can be collected using the CTD water collection system [37,42,43,44,45,46,47,48,49,50,51].

In addition, the amount of sample used for viral research varies. Generally, everyday water contains fewer viral particles, requiring a larger sample for research. In contrast, sewage contains a large number of viral particles, and thus, a smaller sample size is sufficient; approximately 100 mL is enough to detect the abundance of viruses and their genomes [52].

#### 2.1.2. Water Sample Handling and Testing Methods

The various water samples collected are transported to the laboratory at 4 °C and stored at −20 °C until processing. Owing to the large volume of the water samples, the virus particles are dispersed and present in low concentrations, making it necessary to filter and concentrate the water samples to obtain a higher concentration of viruses in suspension before extracting and quantifying the genome. Table 1 summarises the commonly used pore sizes of membranes and concentration methods for filtering water samples. Because viral particles vary in size, the pore sizes of the filter membranes must also differ. Initially, membranes with pore sizes of 1 μm or 2 μm are used to remove large particles, such as sand and gravel, from the water samples. Subsequently, filters with pore sizes of 0.22 μm or 0.45 μm are used to capture viral particles, and membranes with pore sizes of 50 kD or 100 kD are often used at the final stage to capture even smaller virus particles [53,54,55,56]. It is worth noting that, when viruses are filtered using membranes with a pore size of 0.22 µm or 0.45 µm, the filters actually capture the virus particles mainly by electrostatic action, since the pore size of these membranes is much larger than the virus particles [57,58]. Concentration is typically achieved using the tangential flow filtration (TFF) method [59,60,61], FeCl3 concentration [62,63,64], or the PEG (polyethylene glycol) method [40,65,66]. Among these, the TFF method is often used in experiments with large sample volumes and allows for continuous operation. However, it has the disadvantage of being expensive, and for some sensitive biomolecules, the high-speed flow concentration method may affect their activity. The FeCl_3_ concentration method is primarily used for flocculation processes in water treatment, relying on charge neutralisation between the chemical flocculant and the particles. The disadvantages of this method are that it is mainly applicable to water samples, making it unsuitable for all types of samples, and that it may introduce additional ions into the sample, which could affect the purity of the sample if not removed in subsequent steps. The PEG method is currently the most commonly used concentration method in the laboratory. It is simple and effective at concentrating viruses. However, it may require additional treatment to remove excess PEG and salts to minimise their impact on the quality of the sample. The hollow fibre ultrafiltration (HFUF) method can effectively capture and concentrate viruses from water samples, with viral recoveries reaching 70–80% [67,68,69,70]. The celite secondary concentration method after HFUF can effectively increase virus recovery concentration, and the recovered virus concentration can reach about 60% [69].

It has been previously shown that some effluent analyses are conducted following the creation of microcosms, such as the detection of oil contamination using 0.5%, 1%, and 5% diesel oil filtered through a 0.22 μm filter, representing three levels of oil contamination to simulate real-world conditions. These microcosms are incubated at room temperature and manually shaken at regular intervals to simulate tidal fluctuations in surface water [54]. Among the different sources of water samples, the difference in the treatment of glacial samples arises from differences in their form. For example, in the case of some phage DNA enrichment from artificial ice cores from the Tibetan Plateau [71], it is necessary to first melt the ice samples before passing them through filter membranes of different pore sizes. The appropriate method for concentration is then selected, with the FeCl_3_ enrichment technique commonly employed for snow and ice samples [72,73]. Subsequently, DNase I should be added to the viral concentrate to remove any free DNA. The viral genome is then extracted using a commercial kit, followed by amplification and quantification of the viral genome through PCR, qPCR, qRT-PCR, nested-PCR, and the creation of libraries [74,75,76,77,78,79,80]. Since qPCR and RT-qPCR will also directly detect membrane-free nucleic acids, this will make the amount of virus detected under this technique higher than the actual viral abundance [81,82]. To solve this problem, Farkas et al. found a SYBR Green-based RT-qPCR method to accurately quantify viral particles, which could detect 5–5 × 10^4^ viral particle copies [83]. Droplet digital PCR (ddPCR) also has excellent sensitivity, with detection limits of 0.05 pg/μL for viral particle copies [84].

For the detection of the abundance of viral particles in samples, a more standardised method is employed, where a 1–2 mL sub-sample is taken and fixed with methanol/pentanediol [45,46]. However, some literature suggests that methanol/pentanediol fixation may affect the abundance of viruses in the sample [48]. Therefore, viral abundance is determined directly after SYBR Green I staining using either flow cytometry or drop-shot fluorescence microscopy.

### 2.2. Methods for the Study of Viruses in Soil Samples

Current research on soil samples is primarily focused on three types: soil, sediment, and beach sand. The amount of sample required for analysis varies depending on the collection environment, which may affect the level of viral content in the samples. For example, samples collected from beaches with frequent human activity typically require smaller quantities, while those from soil environments with minimal biological activity necessitate larger sample sizes. Viral levels in soil samples typically range from 2.7 × 10^5^ to 4.2 × 10^9^ virus particles/g [47,85,86].

#### 2.2.1. Sampling Methods

Soil samples are generally easier to collect than water samples and can be obtained directly from the surface to 45 cm by a sterile spoon [36,74,75,85,87,88,89,90,91]. Some beach sand samples are collected using a core sampler [92]. To collect sediments from the deep sea, specialised samplers, such as gravity corers and box corers, are required [43,93,94]. The collected soil samples must be sieved using a 2 mm sieve to remove stones and grassroots before conducting further experiments [74,75].

#### 2.2.2. Soil Sample Processing and Testing Methods

As shown in Table 2, there is little variation in the methods used for the filtration, concentration, and determination of the abundance of virus particles in soil samples. However, differences arise in the amount of sample required due to variations in microbial content across different types of samples. For solid samples such as soil, it is usually necessary to recover the supernatant virus particles after resuspension and homogenisation using PBS and then collect the virus particles using different concentration and precipitation methods, among which the TFF method is commonly used to concentrate the supernatant [95] and the PEG method is used to precipitate the virus particles [93,96,97]. This step is followed by DNase treatment to ensure effective purification of the virus particles [77,98,99] before extracting the viral DNA and using a kit for whole-genome amplification and sequencing with the Illumina system. Alternatively, the PBS resuspension step can be omitted, and the entire soil genome can be directly extracted using a soil sample kit. The viral sequence can then be identified post-assembly using VirSorter and DeepVirFinder [91]. Phages that may be present in soil samples are processed by mixing and resuspending the samples in buffer—commonly PBS buffer [100] or sodium citrate buffer [101]—and filtering the samples using a 0.22 μm membrane. The filtered sample solution is then poured into appropriate media, such as lysogeny broth medium (LB medium) or beef digest-based medium (BD medium), for incubation and screening of phage plaques [102]. Phages on the media are usually observed through negative staining transmission electron microscopy [103,104,105]. For example, a method based on magnetic separation and chemiluminescence can be used for rapid detection of *Pseudomonas aeruginosa* phage followed by observation of phage particles by scanning electron microscopy (SEM) and transmission electron microscopy (TEM) [106].

The method for detecting the abundance of virus particles in soil is similar to that used for water samples. This involves staining a sub-sample with SYBR Green I and determining virus abundance using droplet fluorescence microscopy [43,85,107]. Quantification of virus particles in soil samples is typically performed using qPCR or the droplet digital PCR (ddPCR) method [108]. The lowest detection limit can reach 5 fg/μL for ddPCR and 10 fg/μL for qPCR [109].

### 2.3. Methods for Studying Viruses in Aerosol Samples

Aerosol-based samples have been less extensively studied, and it was only after the COVID-19 outbreak that greater attention was given to aerosol samples. Under daily conditions, the concentration of viral particles in the air is approximately 10^3^–10^4^ viral gene copies/m^3^ [110]. In indoor environments, the concentration of viral particles in aerosols can reach 300–700 viral gene copies/m^3^ [93,94,95,96,97,111,112,113,114,115].

#### 2.3.1. Sampling Methods

Aerosol samples are typically collected using either active or passive sampling methods [116]. Passive sampling is mainly based on the natural deposition method, which generally requires prolonged exposure to the environment to collect indoor air [117]. This method has the advantages of ease of use and low cost. However, its disadvantages are the need for a clean sampling environment and poor stability [106]. If fewer particles in the air cause the particles in the collector to bounce back, the data from the passive sampler are inaccurate [118]. Active sampling methods can be classified into solid impact methods, liquid impact methods, centrifugal and cyclone methods, electrostatic precipitation methods, and filtration methods. Among these, the solid impact method is highly sensitive and widely used, though it is complex to operate and requires stringent conditions for the sampling media [119,120]. The liquid impaction method, which minimises microbial damage and operates at a high flow rate, is not suitable for low temperatures and short-duration sampling [121,122]. Centrifugation and cyclone methods are easy to operate, compact, portable, controllable, and low-cost, but they may lead to microbial loss [123,124,125]. Electrostatic precipitation methods help maintain the biostability and viability of samples while enabling the sampling of particles with a wide range of diameters, but they have a limited collection range and a notable impact on the sampling environment [126,127]. Filtration methods, while involving easy-to-carry and low-cost devices, are highly influenced by the materials used and can be complicated to operate [128,129,130]. The collection of aerosol samples typically requires specialised aerosol collectors [51,131], which are often equipped with filters of various pore sizes (typically 25 mm and 47 mm) to collect aerosol particles of different sizes [132].

#### 2.3.2. Aerosol Sample Handling and Detection Methods

For the enrichment of viral particles, the filter membrane is typically resuspended in PBS buffer and centrifuged by filtration. Since viral particles in aerosols are relatively small in diameter, the solution is usually passed through a 0.22 μm filter membrane to remove macromolecules other than viral particles [133]. Viral particles are concentrated using tangential flow filtration (TFF), and the virus concentrations in the samples are detected using RT-PCR, quantitative fluorescent PCR, and other methods after extracting the viral genome with a kit [36,134,135,136]. Currently, for the detection of pathogenic microorganisms in aerosols, simple amplification-based detection (SAMBA) and LAMP ASSAYS are commonly used, which complement the accuracy and sensitivity of the traditional RT-PCR method and reduce the assay time [137]. RT-qPCR was used for the detection of COVID-19. Although RT-qPCR can be very sensitive to RNA in small sample sizes, this method is not widely used due to the high risk of RNA virus infection and the high cost of detection instruments [116]. In addition, the emergence of electrowetting-on-dielectric (EWOD)-based digital microfluidics (DMF) allows for rapid and efficient specific virus detection in a shorter time and with a lower sample volume, and the lowest detection limit for the detection of phage viruses can reach 106 pfu/ml [138].

### 2.4. Methods for the Study of Viruses in Faeces

Research on faecal samples has also increased in recent years, but their processing and detection methods are largely similar to those used for soil samples. Viral gene copies in faecal samples are typically quantified using Qubit concentrations, which range from 80.14 to 98.91 ng/mL [139]. Additionally, viral gene copies in the range of 10^8^ to 10^13^ per gram can be detected in the faeces of patients with gastrointestinal diseases, such as diarrhoea [140,141].

#### 2.4.1. Sampling Methods

Faecal samples are collected using a single method, either with a specialised faecal collector or directly into a sterile bag, ensuring careful measures are taken to avoid contamination during collection. The sampling method for faecal samples does not notably differ between human and animal faeces. Generally, a sample size of 100 mg is required for the study of viral particles in faeces [142,143].

#### 2.4.2. Faecal Sample Processing and Testing Methods

Faecal samples are typically first resuspended in PBS solution [144], and in some studies, the viral genome is extracted from the samples using kits following pre-treatment with antibiotics in PBS solution [145]. Additionally, the resuspension solution is filtered through 0.8 μm and 0.45 μm membranes after the addition of antibiotics and treatment, effectively removing excess macromolecules [139]. RNA viruses are more commonly studied in faecal samples, and viral RNA extraction kits are commonly used to extract the viral RNA, which is then analysed using methods such as RT-qPCR, real-time RT-PCR, and nested RT-PCR and sequenced on the Illumina platform [139,142,145,146,147,148,149]. DNA viruses are generally extracted directly from samples using viral DNA extraction kits and subjected to PCR analysis and sequencing. The difference in the analysis of DNA and RNA viruses is that RNA viruses undergo reverse transcription to cDNA before PCR analysis. A novel real-time RT-PCR method (P-sg-QPCR) has also emerged for the rapid diagnosis and quantification of coronaviruses in the faeces of biological pathogens (e.g., human, cat, canine, porcine, bovine, murine, and avian), which combines the sensitivity of the primer-probe-energy transfer (PriProET) technique [150,151] to allow for faster detection and quantification of viral mutants, with a detection limit of 3.7 × 10^7^ copies/μL [152].

### 2.5. Methods for the Study of Viruses in Plant and Animal Tissues

Current research on plant viruses is relatively straightforward, and the methods for sample collection are the same, involving the direct collection of diseased plant parts for analysis. The viral content in these samples generally ranges from 30 to 3000 ng/mL [153].

The study of viruses in animals has gained increasing attention, especially following the COVID-19 outbreak, heightening concerns about the presence of viruses in animals. However, the handling of animal samples presents challenges, requiring compliance with regulations such as the Laboratory Animal Welfare Act, which mandates that laboratory animals not be abused or killed indiscriminately. In general, virus quantification in animals is typically conducted by directly quantifying antibodies in plasma samples [154,155,156], with viral gene copy abundance reaching up to 5 × 10^6^ copies/μL [157,158,159]. However, not all tests of viral abundance can be replaced by tests of the antibody content in them. When the concentration of antibodies is not sufficient to neutralise the virus, it increases the load of pathogens in it, a phenomenon known as antibody-dependent enhancement (ADE) [160].

#### 2.5.1. Sampling Methods

Because plant viruses are highly visible, their collection is typically straightforward and can be conducted directly at the site of the disease. Generally, a sample size of 2–40 g is required for extracting the viral genome [161,162]. In contrast, the collection of animal samples is typically more complex. It requires careful management to prevent cross-contamination among experimental animals during breeding at the same time. The type of samples often collected from animals include serum, nasal swabs, and oral swabs [163,164,165].

#### 2.5.2. Animal and Plant Sample Handling and Testing Methods

Before extracting viral particles from plant and animal tissues, the samples must be ground and homogenised, after which, the supernatant is collected through centrifugation and filtration. Particularly, for certain viruses extracted from animal tissues such as fish, the samples need to be cultured before homogenisation. Following this, the samples are filtered and screened for viruses. For example, the RNA virus neuron necrosis virus (NNV) extracted from marine fish and shellfish [166], influenza A viruses (IVAs) extracted from bronchial-associated lymphoid tissues in severely affected areas of seal lungs [167], and viral haemorrhagic septicaemia virus (VHSV) extracted from European rainbow trout were isolated directly using cell culture [168]. However, not all viral particle extractions from animals require culturing the tissues. For example, *Redondoviridae* from human bronchial alveoli (BAL) can be directly extracted from the sample using genome amplification kits to obtain the virus’s genome sequence [169].

Most viruses present in plant samples are dsRNA, a stable form of RNA that can be easily extracted from plants. Typically, 10–40 g [170] or 2–5 g of plant tissue is taken for homogenisation. dsRNA extraction is performed using either reagents prepared as described in the literature or commercially available kits. The extracted dsRNA is subsequently analysed by RT-PCR, qPCR, real-time PCR, and multiplexed PCR and sequenced on the Illumina platform [162,171,172,173,174,175,176,177]. High-throughput sequencing is subsequently used to detect and characterise known and unknown plant viruses [178,179,180,181,182,183]. In addition to this method of viral genome identification after the extraction of all genomes in a sample, some earlier studies have employed double antibody sandwich enzyme-linked immunosorbent assay (DAS-ELISA) [184], followed by RT-PCR of selected positive samples. This approach involves resuspending samples in PBS buffer with sodium azide as a preservative, followed by extraction of the plant viral genome and purification using commonly used methods. The sample solution is then subjected to rate-banded sucrose density gradient centrifugation to produce antiserum and measure viral content [185,186]. The cetyltrimethylammonium bromide (CTAB) method is also commonly used to extract viruses from plants. Typically, this process involves homogenising 100 mg of sample in CBTA buffer, followed by centrifugation and RNA extraction. Viral abundance is then assessed using methods such as RT-PCR, LAMP, or RT-ddPCR [187]. For animal samples, suitable tissue sections or serum samples (200 μL) are usually collected. Before extracting the genome from virus-infected tissues, the samples must be homogenised. The tissues are then extracted directly using virus kits, and the genome is analysed through various methods such as PCR and RT-PCR [165,188,189,190,191]. Common quantification methods for viruses in these samples include quantification kits, ELISA, chromatography, RT-PCR, and ddPCR [154,192,193]. The LAMP assay, which is commonly used for virus detection in plant and animal samples, is capable of detecting viral RNA in samples diluted to a concentration of 1 × 10^−7^ ng/μL, and the detection limit of the RT-PCR assay can reach 1 × 10^−4^ ng/μL [194].

## 3. Methods for Concentration of Viral Particles in Different Sample Types and Their Advantages and Disadvantages

In some of the early studies, it was shown that concentration and enrichment methods for viral particles in viral suspension were usually for small-scale techniques with small sample sizes, such as aqueous polymer two-phase separation [195,196], hydroextraction [197], soluble alginate ultrafilter membranes [198], and ultracentrifugation [199,200]. For some experiments with large sample sizes, they were treated with precipitable salts, Fe oxides, polyelectrolytes [201,202,203], and cotton gauze fibres. Although these methods seem to be helpful for experiments with different sample size requirements, the results of concentrating the viral particles are not as good as expected, and many of the methods result in the loss of viral particles in the samples or enrichment of other toxic substances in some water samples instead of accurately obtaining the specific viral particles required for the experiments. Since these methods are not applicable to all virus types, they have been improved based on this situation and started to use positive and negative electrosorption-elution methods with different pore sizes and materials [204,205,206,207,208,209,210,211,212,213]. However, these methods are susceptible to the effects of pH, charge ions, etc., leading to clogging of the filter, which affects the enrichment of virus particles. In order to solve the problem of electrostatic interactions of virus particles, people have switched to ultrafiltration for concentration, which is based on the size of the virus particles and the pore size of the filter membrane, including the TFF tangential flow method commonly used to better solve the problem of membrane blockage [214,215]. In recent years, a combination of ultracentrifugation and density gradient centrifugation has been the most common method of concentration [200,216,217]. This secondary concentration method can greatly increase the recovery of virus particles compared to other concentration methods.

As can be seen from Table 3, ultracentrifugation is one of the most commonly used and highly recovered methods for concentrating virus particles, which is less restrictive to use and has a high rate. Although the two-step concentration method can also obtain a higher concentration of virus particle recovery solution, the steps are complicated. The greatest advantage of the two-step concentration method over ultracentrifugation is that it can be used at outdoor sampling sites where electricity is available and does not have to be confined to a laboratory environment.

## 4. Methods for Quantification of Viral Particles in Different Sample Types and Their Advantages and Disadvantages

Currently, different collection, processing, and quantification methods are employed for various types of samples studied in the current habitat. In general, water samples are collected directly using sterile bags, bottles, or CTD water collection system. Snow, ice, and soil samples are collected directly with sterile shovels and then placed into sterile sampling bags or bottles. Faecal samples and aerosols require specialised sampling equipment. Plant samples are collected by selecting representative specimens from uncontaminated areas, and the site of investigation is collected using sterile tools, placing them into sampling bags. Animal samples are typically collected from appropriate tissues or body fluids.

The detection of virus abundance using flow cytometry is widely applied to water samples, though the method has some limitations. Flow cytometry may not remove enough interfering particles compared to drop-shot fluorescence microscopy, resulting in inflated virus particle abundance results [85]. The quantitative detection of virus particles in soil samples typically employs qPCR or ddPCR technology. ddPCR is generally more sensitive to virus particles at lower target concentrations, and some studies have shown that ddPCR offers superior accuracy, reproducibility, sensitivity, and stability for the quantification of bacteria and fungi compared to qPCR, resulting in more accurate outcomes [109]. However, the ddPCR method also has drawbacks, such as high cost, complex protocols, and a limited detection range; thus, it can be used as a complementary method to qPCR method [229]. Stool samples, which usually contain a high concentration of viral particles, generally do not require a highly sensitive detection method. As a result, qPCR, RT-PCR, or RT-qPCR is commonly selected for quantification [230,231,232,233]. For studies on human clinical samples, methods such as the TaqMan Array Card (TAC) assay [234] and the LAMP assay are used for the simultaneous detection of multiple enteric pathogens. Virus quantification in plant samples frequently utilises serological assays, such as the DAS-ELISA, as well as qPCR, RT-PCR, RT-qPCR, and RT-ddPCR [109,235,236,237,238,239]. DAS-ELISA is commonly used to detect tomato spotted wilt orthotropic virus (TSWV) in peanut leaves. However, the TSWV load detected by DAS-ELISA in the inter-root of asymptomatic peanut leaves has been found to be significantly higher than that detected by other techniques, suggesting that DAS-ELISA may overestimate TSWV viral loads [240]. For the quantification of citrus tartar leaf virus (CTLV), the RT-ddPCR method is used, as it can be up to 10-fold more sensitive than RT-PCR [158]. Furthermore, the LAMP assay has been demonstrated to be more sensitive than RT-PCR and specific for the detection of ToMV. RT-PCR is often used in the quantification of viruses in animal serum samples, although differences exist in this assay. The RT-PCR method can be divided into one-step and two-step protocols, with the one-step protocol offering a higher limit of detection of viral load and greater sensitivity [241,242], whereas the two-step RT-PCR reaction provides greater flexibility and allows for better optimisation [188].

Table 4 lists the currently used methods for quantitative detection of virus particles. It can be seen that RT-qPCR has the lowest detection limit, which is also the most used method at present. There are also some improved methods based on RT-qPCR and ddPCR that can quantify the viral copy number more accurately. Currently, there is also a complex PCR method, epicPCR, which is usually amplified with specific primers and then quantified using common PCR methods such as qPCR. epicPCR can rapidly identify specific viral targets in situ and reveal virus–host relationships in a non-culture manner. The method is highly efficient with low equipment requirements but suffers from some primer bias [243,244].

## 5. Outlook

The study of viruses in environmental habitats currently faces several significant challenges, including the low concentration of viral content in samples, high detection limits, and operational complexity. These challenges limit high-throughput processing and rapid response capabilities in virus detection, both for outbreak surveillance and environmental virology studies. Although the development of genome sequencing technology has advanced rapidly, the pre-processing steps for samples remain complex. In future research, the enrichment methods for virus particles can be optimised to improve the recovery rate of the virus and the sensitivity of detection. Additionally, the development of automated equipment for sample processing could reduce manual labour, increase processing speed, and effectively reduce human contamination at the same time. Interdisciplinary approaches, incorporating molecular biology and bioinformatics, could also help create comprehensive virus detection platforms and enable real-time monitoring technology for virus particles in environmental samples, facilitating rapid responses to public health emergencies.

These experimental challenges that have emerged in the past two years along with the limitations of objective factors such as experimental samples and the subjective aspects of unavoidable human contamination have made the study of pathogenic microorganisms complex. Additionally, the ultrafiltration and concentration steps involved in enriching pathogenic microorganisms and other steps of the process require extended experimental time and stringent experimental conditions, further complicating the study of pathogenic microorganisms. For example, during field sampling, there is a high probability that the samples will be contaminated with environmental pollutants that may affect the enrichment of the target virus particles. When researchers handle the samples in the laboratory, the samples may be contaminated due to operator error, or errors in the selection of methods and quantification instruments may lead to inaccuracies in the study of viral particles. Currently, there is no effective and convenient method to enrich and study pathogenic microorganisms with high quality and high concentration. We can predict the trend of rapid methods by summarising the advantages of some of the current emerging methods, including allowing for high throughput, automation, low cost, objectivity, reproducibility, high precision and accuracy, and broad applicability, which provides a direction for the development of more advanced viral titre assays in the future.

## 6. Conclusions

This review summarises the methods used in the literature to enrich and extract pathogenic microorganisms from various environments. Despite the differences in these environments, the methods for the enrichment and extraction of pathogenic microorganisms are broadly similar, with differences only occurring in the processing of the samples. However, for the detection of pathogenic microorganisms, the microorganisms must first be concentrated, followed by the extraction of genetic material using kits. Although the methods are relatively uniform, several challenges remain in the study of pathogenic microorganisms. These include the low concentration of pathogenic microorganisms in environmental samples, contamination introduced during sample enrichment and concentration due to the experimental environment and procedures, and the difficulty in extracting pathogenic gene sequences owing to the low abundance of microorganisms in the samples. Additionally, environmental factors, such as differences in sample collection conditions and experimental temperatures, can induce morphological changes in the samples, complicating the analysis further. These morphological changes can also influence both the quantity and survival rate of pathogenic microorganisms in the samples.

## Figures and Tables

**Table 1:** Collection and treatment of different water samples.

Sample Source	Method and Processing	Sample Size Required	Filtration	Concentration	Detection	Virus Abundance Test
Surface water, tap water, water for recreational purposes	Directly sampling or using an electric pump with a sterile sampling bag or sterile sampling bottle	1000 L or more	1 μm or 2 μm membrane. 0.45 μm or 0.22 μm membrane. 50 kDa or 30 kDa membrane.	TFF method, density gradient centrifugation in ultra-high-speed centrifuge, aluminium salt coagulation precipitation, and the PEG method	Illumina sequencing, PCR, qPCR, RT-qPCR	1–2 mL sub-samples are dispensed and fixed in methanol/pentanediol or stained directly with SYBR Green I; viral abundance is determined by drop-shot fluorescence microscopy
Deep water	CTD temperature and Salt Depth Profiler with water collection system to collect water samples in sterile bottles	1000 L or more	1 μm or 2 μm membrane. 0.45 μm or 0.22 μm membrane. 100 kDa or 50 kDa membrane.		Illumina sequencing, PCR, RAPD-PCR, RT-qPCR	
Sewage	Sterile sampling bottle; some oil contamination studies need to prepare microcosms in advance	100 mL or more	1 μm or 2 μm membrane. 0.45 μm or 0.22 μm membrane. 100 kDa or 50 kDa membrane.		Illumina sequencing, PCR, qPCR, RT-qPCR, nest-PCR	
Glacial water	Sterile bag and 10 L acid-washed polycarbonate bottle	10 L or more	20 μm, 1 μm, 0.22 μm.	Aluminium salt coagulation precipitation (FeCl_3_ flocculation)	Illumina sequencing, PCR	

**Table 2:** Collection and treatment of different soil samples.

Sample Source	Method and Processing	Sample Size Required	Filtration	Concentration	Detection	Virus Abundance Test
Soil	Sterile sampling bag, sterile sampling bottle	30–600 g	PBS resuspension, 0.22 μm membrane filtration	TFF method, density gradient centrifugation in ultra-high-speed centrifuge, aluminium salt coagulation precipitation, and the PEG method	Illumina sequencing, PCR, qPCR	1–2 mL sub-samples are dispensed and fixed in methanol/pentanediol or stained directly with SYBR Green I; viral abundance is determined by drop-shot fluorescence microscopy
Sediment	Sterile sampling bag	5–50 g			Illumina sequencing, PCR, nest-PCR, RT-PCR	
Beach sand	Sterile sampling bag, sterile sampling bottle	10–50 g			Illumina sequencing, PCR, qPCR, RT-PCR, HT-qPCR	

**Table 3:** Comparison of commonly used virus particle concentration methods.

Concentration Methods	Virus Particle Recovery (%)	Advantages and Disadvantages	Reference
Ultracentrifugation	9.1–73%	High recycling rate, specialised equipment	[218,219,220,221,222]
PEG	0.08–52.8%	Large sample size, low abundance of viruses, low cost but time-consuming	[223,224,225]
Novel Nanotrap Microbiome Particles (NMP)	0.4–21%	Fast and less time-consuming, small sample size but imprecise	[226]
Membrane adsorption	0.7–56%	Specialised equipment, lower availability	[220,227]
TFF	23.8–42.5%	Separated by size, not easy to clog, more residual contaminated protein	[228]
HFUF + Celite secondary concentration	60% or so	High recovery rate, low environmental requirements but complicated steps, can not handle dirty samples	[69]

**Table 4:** Comparison of commonly used quantification methods for virus particles.

Quantitative Methods	Quantitative Detection Limit (Copies/μL)	Advantages and Disadvantages	Reference
RT-qPCR	10^2^–10^6^	Detects relative titres, wider quantitative range, and lower limit of detection, requires specialised instrumentation	[238,245,246,247]
ddPCR	10^3^–10^7^	Low detection limit and high sensitivity	[248]
LAMP	105	Easy to use, fast, low cost, high limit of detection	[249]
DAS-ELISA	109	Large sample size, long time required, poor sensitivity	[238,250,251]
qPCR	10^2^–10^6^	Low cost, simple operation, wide range of applications, weak specificity, inaccurate results	[252]
